# Impact of Digital Safety Plan Activation on Subsequent Emergency Departments Visits Following an Initial Suicide Attempt: Quasi-Experimental Study

**DOI:** 10.2196/70253

**Published:** 2025-06-17

**Authors:** María Luisa Barrigón, Carlos Schmidt, Matilde Elices, Alejandro Porras-Segovia, Ana María De Granda-Beltrán, Antonio Artés-Rodríguez, Philippe Courtet, Víctor Pérez-Sola, Enrique Baca-García

**Affiliations:** 1 Institute of Psychiatry and Mental Health, Hospital General Universitario Gregorio Marañón, IiSGM, School of Medicine, Universidad Complutense de Madrid, Madrid, Spain; 2 Centro de Investigación Biomédica en Red (CIBERSAM), Madrid, Spain; 3 Hospital del Mar Research Institute (IMIM), Dr. Aiguader 88, Barcelona, 08003, Spain, 34 671377507; 4 Fundacion Jiménez Díaz Health Research Institute, Department of Psychiatry, Rey Juan Carlos Hospital Móstoles, Madrid, Spain; 5 Department of Psychiatry, Fundacion Jiménez Díaz University Hospital, Madrid, Spain; 6 Department of Signal Theory and Communications, Universidad Carlos III de Madrid, Leganés, Madrid, Spain; 7 Evidence-Based Behavior (eB2), Madrid, Spain; 8 Instituto de Investigación Sanitaria Gregorio Marañón, Madrid, Spain; 9 Department of Emergency Psychiatry and Acute Care, Centre Hospitalier Universitaire Montpellier, University of Montpellier, Montpellier, France; 10 Universidad Pompeu Fabra (UPF), Barcelona, Spain; 11 Departamento de Psiquiatría, Universidad Autónoma de Madrid, Madrid, Spain; 12 Departamento de Psiquiatría, Hospital Central de Villalba Villalba, Madrid, Spain; 13 Departamento de Psiquiatría, Hospital Universitario Infanta Elena Valdemoro, Madrid, Spain

**Keywords:** suicide prevention, digital safety plan, brief interventions, emergency department, secondary suicide prevention, suicidal behavior

## Abstract

**Background:**

Suicide is a significant global public health concern. Individuals with suicidal behaviors often seek help in emergency departments (ED), making mental health providers critical to suicide prevention. Brief interventions such as safety planning are essential in these settings. However, there is a limited understanding of how mobile digital safety planning apps can aid in secondary suicide prevention.

**Objective:**

This study evaluated the effectiveness of a digital safety plan, delivered through the *MeMind* app, in reducing ED visits associated with suicidal behavior (ie, suicidal ideation or attempt).

**Methods:**

A one-year follow-up was conducted for individuals who presented to the ED for an index event of suicidal behavior (N=78). Participants were provided with a digital safety plan on their mobile devices and instructed to activate it during future suicidal crises.

**Results:**

At follow-up, participants who activated the digital safety plan showed a 50% lower likelihood of returning to the ED, when compared to those who did not activate it.

**Conclusions:**

These findings suggest that digital safety planning may serve as a scalable and accessible intervention with the potential to significantly contribute to suicide prevention efforts.

## Introduction

Suicidal behavior is a complex and multifactorial phenomenon that constitutes a public health concern. The World Health Organization estimates that over 700,000 individuals die by suicide annually, with data suggesting that there are more than 20 attempted suicides for each suicide-related death [1,2]. Globally, the suicide rate is estimated at 9.4 per 100,000 people, particularly among those aged 15 to 34 years, with a higher prevalence in men compared to women [3]. In Spain, the estimated suicide rate is 8.4 per 100,000 people, representing the second leading cause of non-natural death by external causes, with approximately 11 suicides occurring daily [4].

The consequences of suicidal behavior have a significant impact on individuals, families, and the society, emphasizing the urgent need for health systems to prioritize suicide prevention and implement effective strategies [5]. However, there remains a need to enhance the effectiveness of current suicide interventions, particularly for individuals presenting to emergency departments (ED) and those hospitalized due to suicidal ideation or behaviors, who exhibit a high risk of reattempting suicide within the initial months’ post discharge [6]. This increased risk of reattempt is further amplified when individuals have a history of suicide attempts and a diagnosed psychiatric disorder [7].

Studies indicate that face-to-face psychological interventions are effective in reducing suicidal behavior compared to treatment-as-usual. However, the anticipated outcomes are primarily observed in intensive and long-term intervention approaches [8]. Conversely, there is a growing interest in digital and brief interventions for managing suicidal behavior, driven by their cost-effectiveness and relevance when immediate access to mental health care is limited [9]. Brief interventions, administered by trained professionals in acute care settings, aim to provide tools for managing suicidal crises and reduce suicide risk and have demonstrated effectiveness in reducing subsequent suicide attempts [10].

Among brief interventions for suicide prevention, safety planning intervention (SPI) is commonly used [11]. SPI is a brief therapeutic practice, typically lasting 20‐45 minutes, aimed at reducing the imminent risk of suicidal behavior. This is accomplished through a collaborative process between clinicians and patients, where a personalized plan is developed. This plan guides individuals to identify their thoughts, feelings, and experiences immediately preceding a crisis, and establishing coping strategies and activation of support resources upon the recurrence of suicidal thoughts. SPI includes personal warning signs, strategies for self-management, social support, emergency contacts, reasons for living, and measures to restrict access to lethal means [12]. Systematic reviews support the effectiveness of traditional SPI in reducing suicidal behaviors [10,11,13] with studies showing that SPI can decrease suicidal behaviors by up to 45% [14].

Safety planning is framed within a participatory care model, which emphasizes active collaboration between clinicians and patients to co-design personalized strategies for managing suicidal crises. This model fosters patient empowerment, shared decision-making, tailored interventions, and enhances patient satisfaction, improves treatment adherence, and reduces costs [15]. This participatory care model has been effectively applied in several suicide-specific interventions [16].

Despite the potential benefits of safety planning in reducing suicidal behavior, limited information exists regarding its usability outside of treatment settings. For example, it has been observed that individuals seeking help for suicide-related concerns are largely unaware of safety planning or have not incorporated it into their treatment plans [17]. Additional barriers related to safety plan usage include its implementation in the presence of depressive mood, particularly in the absence of collaborative development with a clinician [18]. Furthermore, as traditional SPIs are often paper-based, access barriers may arise during crisis situations due to difficulties in retrieving the information.

Digital safety planning via mobile-apps offers a viable alternative to overcome these limitations by enhancing immediate and ubiquitous access through personal mobile devices and providing a more dynamic and user-friendly platform. Through such apps, several functions can be incorporated to enable access to videos, photos, import of significant contacts, or location-based access to emergency services [19]. For instance, individuals can integrate family photos or directly access contact information for significant support persons or health care professionals or centers, potentially triggering coping strategies related to suicide more directly and effectively. These strategies embody a participatory care model, actively engaging patients in their own treatment [20]. Moreover, the use of an ecological momentary intervention (EMI) through mobile apps facilitates real-time monitoring and usability tracking of the digital safety plan [21]. Such data collection can provide insights into who uses the safety plan, when, and which strategies are most beneficial.

Previous studies have shown that mobile-app based safety plans demonstrate high acceptability and feasibility, although evidence regarding their effectiveness is still limited [19]. To date, only a few studies have evaluated the effectiveness of a digital safety plan. Two pilot studies found that the use of a digital safety plan reduced suicidal ideation [22,23] and a subsequent study with a larger sample found that the use of a digital safety plan increased suicide-related coping, which was associated with a reduction in suicidal ideation. Further, greater perceived utility of the digital safety plan (eg, by using customizable content) was associated with higher suicide-related coping [24]. However, the follow-up period in these studies was limited to three months, thereby precluding an evaluation of the long-term efficacy of digital safety plans or their ability to reduce in emergency department (ED) visits due to recurrent suicidal behavior.

In this study, a one-year follow-up was conducted among individuals who presented to the ED with suicidal behavior (ie, suicidal ideation or attempt). Participants were provided with a digital safety plan on their mobile devices and instructed to activate it during future suicidal crises. The study aimed to assess whether activating the digital safety plan reduced subsequent ED visits associated with suicidal behavior.

## Methods

A quasi-experimental design and an EMI were employed to monitor the use and activation of a digital safety plan through the *MeMind* app [25].

### Participants

Data were collected in the EDs of two hospitals in Madrid, Spain, between September 2022 and August 2023. Participants were adults (aged ≥18 years) presenting to the ED for suicidal behavior. Suicidal behavior was defined as any action or self-injurious act with the intent to die, encompassing both suicidal ideation and suicide attempts. Once potential participants received standard emergency care and were deemed medically stable, they were offered the opportunity to participate in the study prior to discharge. Those who consented to participate met with a psychiatrist for 30‐40 minutes to receive a detailed explanation of the study, install the study app on their smartphones, and complete the digital safety plan. The inclusion criteria were (1) presentation to the ED for suicidal behavior; (2) ownership of a smartphone with internet connection; (3) speaking and understanding Spanish; and (4) being able to give informed consent. Exclusion criteria included unwillingness to install the app or limited smartphone access.

### Measures

#### Digital Safety Plan

**Figure 1.**
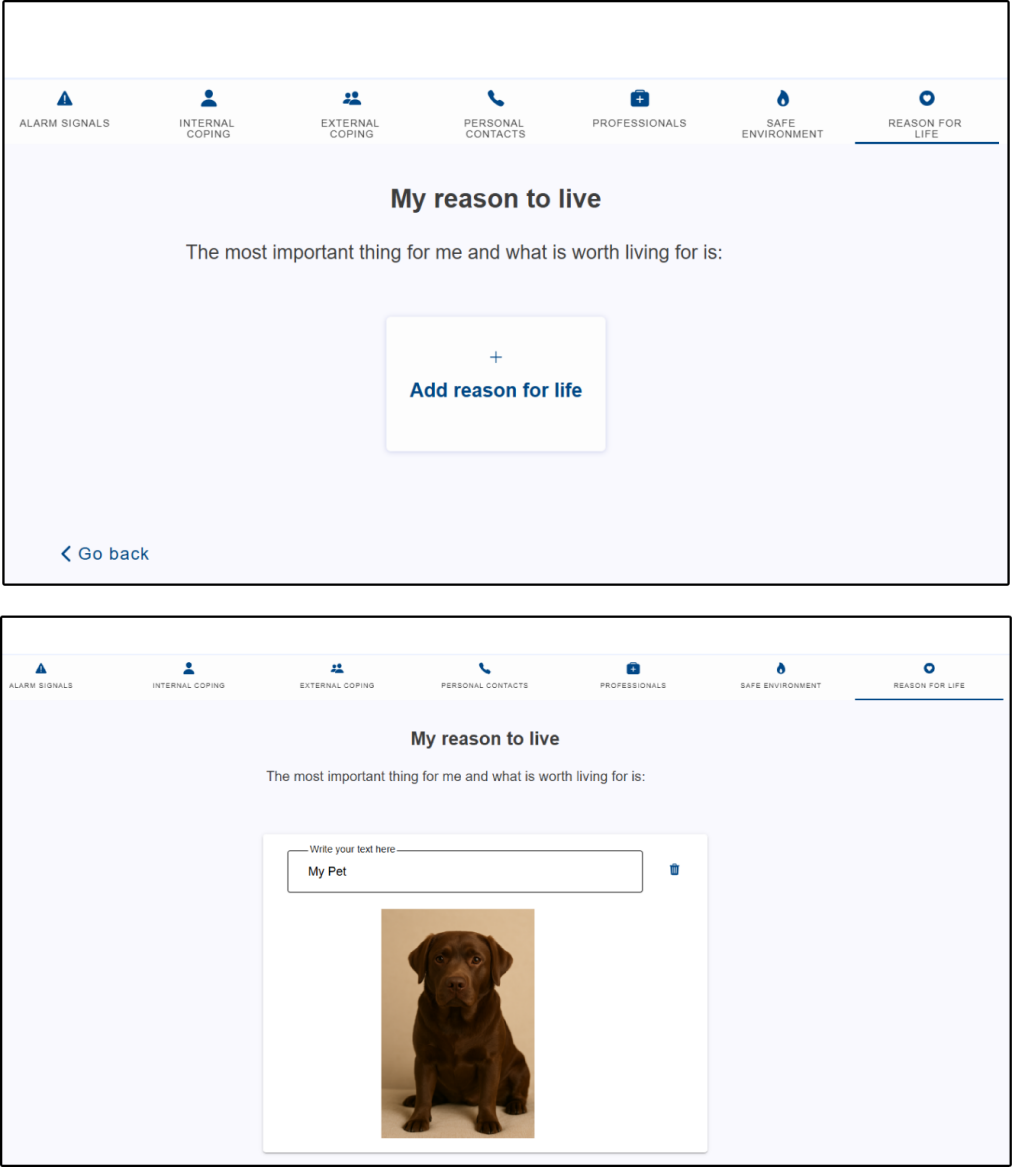
Example of MeMind app safety plan configuration; artificial intelligence-generated image, in response to the request "Generate an image of a Labrador Retriever as a companion dog" (Generator: Copilot, Microsoft; June 11, 2025; Requestor: Carlos Schmidt) [27].

Participants received access to a digital safety plan for suicidal crises through the *MeMind* app [25] (Figure 1). *MeMind* is designed to be compatible with Android and iOS operating systems and can be downloaded for free on Google Play and the Apple App Store. After installation, participants collaborated with psychiatrists to configure the plan, which included seven customizable tabs: warning signs, internal and external coping strategies, personal and professional contacts, safe environments, and reasons to live. The app, which was validated in previous studies demonstrated good feasibility and acceptability [26]. Participants were instructed to activate the plan during subsequent suicidal crises. Data on digital safety plan configuration and activation were collected over a one-year follow-up. Specifically, the frequency of digital safety plan configuration and activation of each of the seven incorporated tabs or functions was recorded. Configuration allowed participants to use the “Configure Safety Plan” feature, enabling them to review or add new information to each tab or function outside of crisis periods. Activation involved using an in-app feature labeled “Activate Safety Plan,” which provided access to the information within the seven previously configured tabs or functions aimed at implementing coping strategies during suicidal crises. Participants were encouraged to configure the digital safety plan as needed and to activate it during crises triggered by the onset of suicidal behavior.

#### Suicidal Behavior

Baseline assessments used the Spanish version of the Columbia Suicide Severity Rating Scale (C-SSRS) [28]. The C-SSRS is a semi-structured interview that evaluates the occurrence, severity, and frequency of suicidal ideation and behavior [29]. Information on the severity of ideation (most severe ideation, intensity, frequency, duration, and controllability) and previous history of suicide attempts was collected.

#### Statistical Analyses

A Cox proportional hazards model with random effects was used to investigate the association between digital safety plan activation and subsequent ED visits for suicidal behavior (ie, suicidal ideation or attempt) during the one-year follow-up. The outcome variable was ED visits for suicidal behavior (yes vs no), with time measured in days over a 365-day follow-up period. The primary predictor variable was digital safety plan activation (yes vs no). This analysis assessed whether participants who activated the safety plan during the follow-up period experienced fewer ED visits for suicidal behavior compared to those who did not (ie, quasi-control group). Age and sex were included as covariates. Random effects were specified for each participant, with participant ID and days of digital safety plan use (ie, configuration or activation) as random intercepts. All statistical analyses were conducted using a two-sided significance level of α=0.05. Analyses were conducted using R software (version 1.1.463; R Foundation for Statistical Computing), including the *survival* and *coxme* packages [30-32].

### Ethical Considerations

The study was approved by the Research Ethics Committee of the Fundacion Jimenez Diaz Hospital (Approval number: EC005-21_FJD). All participants provided informed consent. Participants were free to withdraw from the study at any time or decline further participation during follow-up without affecting their medical care. All collected data were anonymized to protect participant identity. No compensation was provided for study participation.

## Results

### Demographic Characteristics

A total of 78 participants were included in the study, with a mean age of 39.08 (SD 13.45) years; most were female (n=56, 72%). According to the baseline suicidal behavior characteristics (ie, C-SSRS), 72 (90%) participants exhibited high-intensity suicidal ideation, and 63 (80%) participants reported prior suicide attempts (Table 1). Over the one-year follow-up, 24 (31%) participants returned to the ED services for suicidal behavior (ie, suicidal ideation or attempt).

**Table 1.** Baseline sociodemographic characteristics of the total sample.

Characteristics	Participants (N=78)
Age (years), mean (SD)	39.08 (13.45)
Sex (female), n (%)	56 (72)
Emergency department visits, n (%)	
Only 1 (index event)	54 (69)
≥2	24 (31)
Suicidality (C-SSRS)^a^	
SI^b^ (yes), n (%)	72 (90)
Intensity of SI^c^, mean (SD; range)	4.10 (1.03; range 1‐5)
Frequency of SI^d^, mean (SD; range)	2.67 (1.24; range 1‐5)
Duration of SI^e^, mean (SD; range)	3.19 (1.37; range 1‐5)
Controllability of SI^f^, mean (SD; range)	3.18 (1.47; range 0‐5)
Previous suicidal attempts (yes), n (%)	63 (81)

a C-SSRS: Columbia Suicide Severity Rating Scale.

b SI: suicidal ideation.

c Intensity of suicidal ideation: severity of ideation, with 1 being the least severe and 5 being the most severe.

d Frequency of SI: “How often have you had these thoughts?“, ranging from (1) less than once a week to (5) many times a day.

e Duration of SI: “When you have those thoughts, how long do they last?” ranging from (1) fleeting/a few seconds or minutes to (5) more than 8 hours/persistent or continuous.

f Controllability SI: “Could/can you stop thinking about killing yourself or wanting to die if you want to?” ranging from (1) You can control the thoughts easily to (5) You cannot control the thoughts, or (0) You do not try to control the thoughts.

Subsequently, the frequency of return to the ED and digital safety plan usability by the participants during the follow-up period were explored. A total of 2.768 observations were collected for the digital safety plan usage. Of the overall enrolled participants, 31 (40%) activated the digital safety plan during the follow-up period.

**Figure 2.**
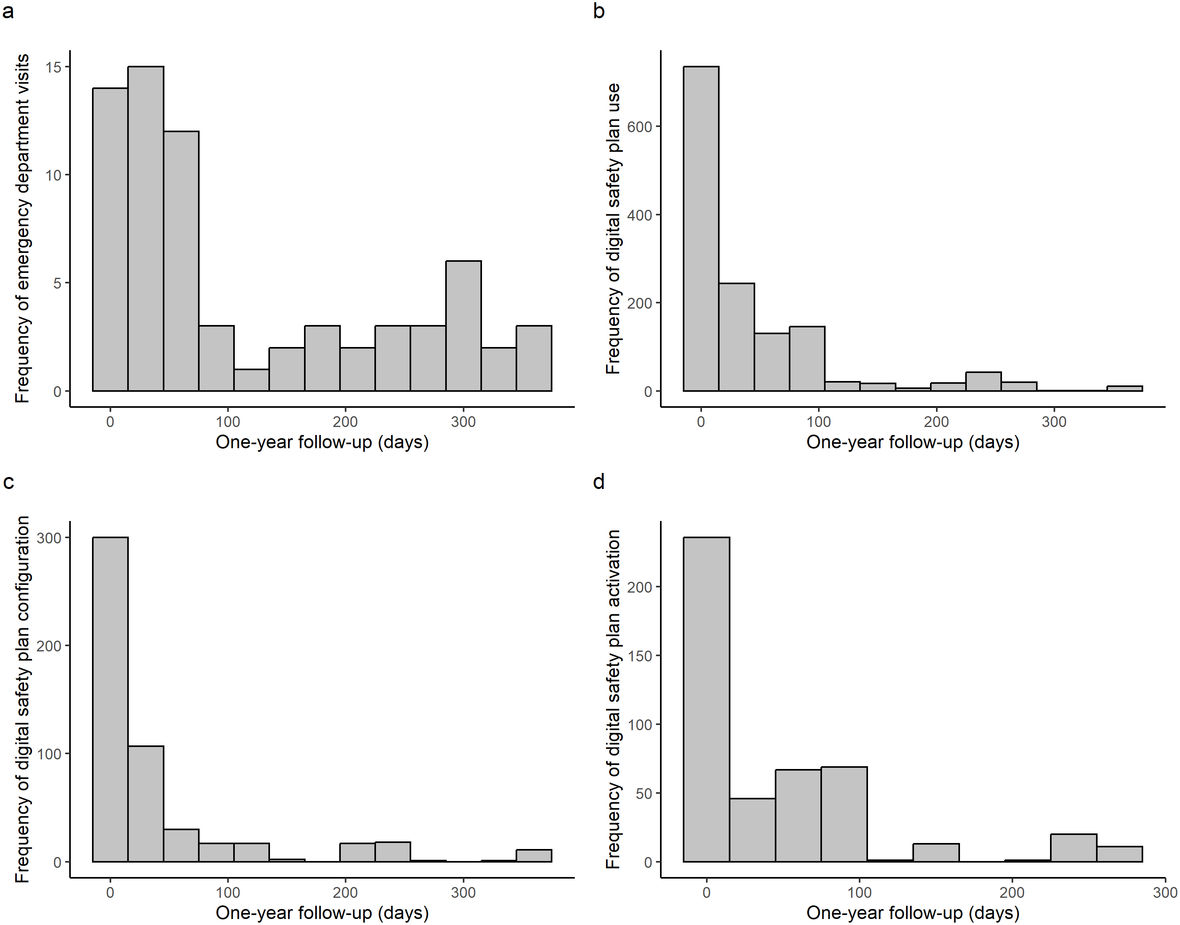
(A) Frequency of emergency department returns due to suicidal behavior; (B) Frequency of total digital safety plan use; (C-D) Configuration and activation of digital safety plan during follow-up.

Given the non-normal distribution and positive skew of these variables, we reported the median (IQR) and 90th percentile (P90). Within the one-year follow-up, the median time for return to the ED postindex event was 55 (IQR 211, range 1‐359; P90=301) days, with a higher frequency observed within the first 60 days, followed by a more uniform distribution throughout the remaining year (Figure 2A). Excluding the initial app installation and collaborative safety plan installation with the psychiatrist, the median overall digital safety plan usage occurred at 14 (IQR 47, range 2‐359; P90=105) days (Figure 2B). When examining the frequency of digital safety plan configuration (ie, personalizing the safety plan), the median usage occurred at 11 (IQR 28, range 2‐359; P90=107) days, with a decreased frequency throughout the rest of the year (Figure 2C). For digital safety plan activation (ie, accessing the safety plan during crisis moments), the median usage occurred at 15 (IQR 67, range 2‐260; P90=112) days, with a stable frequency in the first 90 days and a significantly reduced frequency in the remaining follow-up period (Figure 2D). Overall, 90% (62/69) of participants returned to the ED due to suicidal behavior within 300 days, while 90% of digital safety plan configurations (1392/1547) and activations (529/588) occurred within the first 100 days of follow-up.

When examining the usage of the digital safety plan configuration, “external coping strategies” (n=362, 23.30%), “professional ask for help” (n=328, 21.10%), and “warning signals” (n=234, 15%) were the most frequently accessed functions, followed by “personal contacts” (n=188, 12.10%) and “internal coping strategies” (n=178, 11.40%); “creating a safe environment” (n=141, 9.10%) and “reasons to live” (n=124, 8%) were less frequently used. For digital safety plan activation, the most frequently activated tabs were “external coping strategies” (n=140, 22.90%), “warning signals” (n =125, 20.85%), “professional ask for help” (n= 120, 19.48%), “internal coping strategies” (n =74, 12.30%), and “personal contacts” (n =64, 10.42%); “creating a safe environment” (n= 50, 7.69%) and “reasons to live” (n= 41, 6.32%) were less frequently used. No significant differences were found in age, sex, suicidal behavior characteristics, or severity of suicidal ideation between those who activated the digital safety plan and those who did not (all *P* values >.05).

In general, while all safety plan function or sections were used to some extent, we observed a usage pattern that corresponded with the tab sequence in the app’s interface. Specifically, “creating a safe environment” and “reasons to live,” which were positioned at the end of the app’s interface, were least frequently used. This finding suggests that the arrangement of the functions influenced the usability of the digital safety plan.

### Association Between Digital Safety Plan Use and Subsequent Emergency Department Visits

Participants who activated the digital safety plan had a lower frequency of ED visits compared to those who did not activate the plan (n=102, 16.6% vs n=313, 29.1%; *χ²_1_*=37.48, *P*<.001).

**Figure 3.**
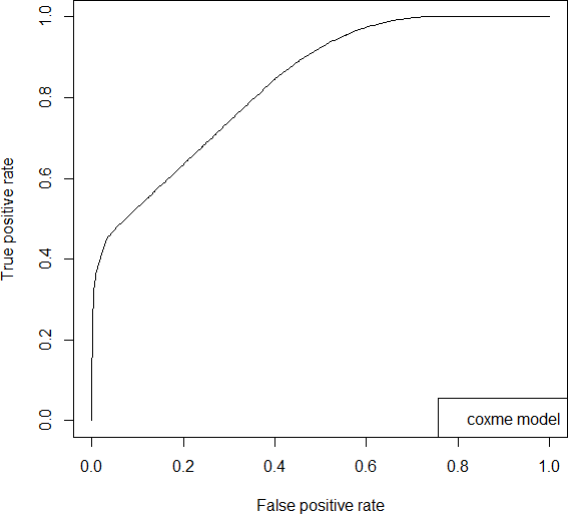
Receiver operating characteristic curve for survival model.

Subsequent analyses examined the predictive utility of digital safety plan activation for ED visits over the one-year follow-up period. Using the *coxme* function, we identified the best-fitting model with participant ID and days of safety plan usage as random effects. Receiver operating characteristic analysis demonstrated good discriminatory capacity of the model (area under the curve [AUC]=0.82) for predicting ED visits for suicidal behavior based on digital safety plan activation (Figure 3).

**Figure 4.**
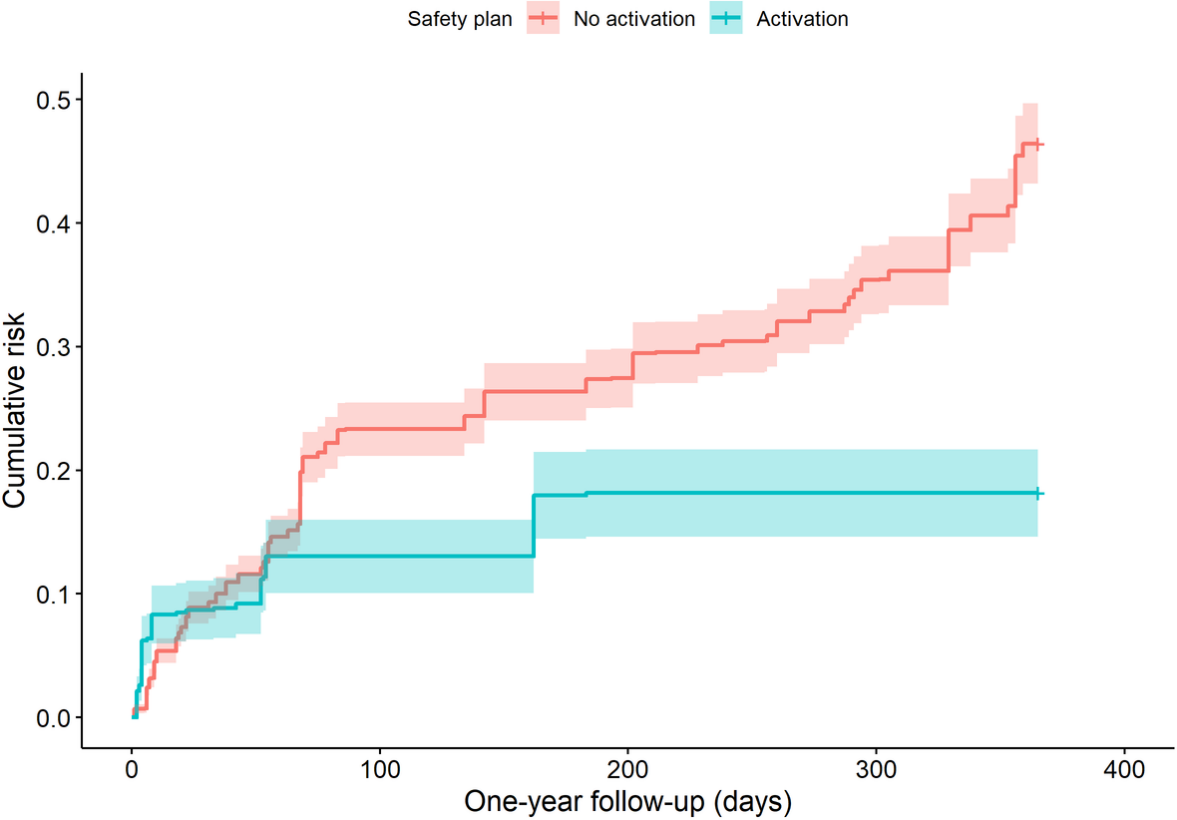
Cumulative risk of emergency department visits for suicidal behavior based on digital safety plan activation.

The Cox proportional hazards model, adjusted for participant demographics, revealed a significant association between digital safety plan activation and a reduced likelihood of subsequent ED visits. Participants who activated the safety plan had a 50% lower likelihood of returning to the ED for suicidal behavior (ie, suicidal ideation or attempt) compared to those who did not (Figure 4). Furthermore, male participants and those aged ≥30 showed a reduced likelihood of returning for subsequent ED visits (Table 2). These results suggest that engaging with the digital safety plan and consequently accessing preconfigured suicide-related coping strategies within the app, may enable individuals to effectively manage suicidal thoughts and prevent escalation during a suicidal crisis, potentially reducing the frequency of ED readmissions. Additionally, sex and age were important clinical factors and should be considered in predicting ED readmissions for suicidality.

**Table 2.** Association between digital safety plan activation and emergency department visits.

Predictors	β (SE)	HR^a^ (95% CI)	*P* value
Safety plan activation			<.001
No (reference)	–	–
Yes	–0.68 (.19)	0.50 (0.34-0.73)	
Sex			.03
Female (reference)	–	–	
Male	–.53 (.26)	.58 (.34-.97)	
Age (years)			
18-29 (reference)	–	–	
30‐49	–.71 (0.26)	–.49 (0.29-0.81)	.006
≥50	–2.01 (0.32)	–.13 (0.07‐025)	<.001

a HR: hazard ratio.

## Discussion

### Principal Findings

Prior studies have established the relevance of safety plan usage in preventing or managing suicidal crises. However, to date, the effectiveness of a mobile digital safety plan in reducing ED visits for suicidal behavior has not been evaluated. This study found that participants who chose to activate a digital safety plan showed a 50% reduction in the likelihood of subsequent suicide-related ED visits, compared to those who did not activate it.

Additionally, male participants and those aged ≥30 were less likely to return to emergency services for suicidal behavior during the one-year follow-up after an index event, compared to female and younger participants. Generally, men are less inclined to seek assistance for suicide-related issues, even though they tend to use more hazardous methods and exhibit elevated fatal-suicide rates globally [3], including in European countries [33]. Nonetheless, no association was observed between digital safety plan activation and participants’ sex, age, or severity of suicidal ideation.

Our analysis also revealed that 30.8% of participants returned to the ED due to suicidal behavior following the index event, predominantly within the first 60 days. The initial three-month period post discharge represents a time of heightened risk for reattempt, thus highlighting the relevance of prevention and follow-up during this phase [6]. Similarly, the highest frequency of digital safety plan activation occurred within the first few weeks, with a median of 15 days. Consequently, digital safety plan usage was brief and strongly associated with the index event. Prior research has indicated that the usability of the digital safety plan is sporadic and concentrated in the early follow-up weeks [24]. Safety plans are designed for use during suicidal crises [12]; therefore, it is expected that users will engage with them only when necessary, particularly at the onset of suicidal thoughts.

The most frequently activated sections of the digital safety plan were “warning signs,” “external coping strategies,” and “professional contacts.” In contrast, less frequently used sections included “reasons to live,” “safe environments,” “personal contacts,” and “internal coping strategies.” These findings suggest a potential preference for seeking external support during suicidal behavior, such as contacting professionals, rather than relying solely on internal resources. This observation is consistent with findings that indicate social support serves as a protective factor against suicidal behavior [34], particularly among individuals with a history of suicide attempts [35]. It is also possible that during periods of intense or severe suicidal ideation, internal coping strategies become more difficult to activate and manage independently, whereas external suicide-related coping are more readily accessible [36]. Additionally, the design and interface of the digital safety plan app may impact which features are prioritized by users. For example, the interface’s tab order—with “warning signs” positioned first and “reasons to live” last—may impact usability. Future research should further explore the usability of the app and identify which sections are most useful to users. Suggested improvements could include in-app digital scales to assess satisfaction with app use and the perceived effectiveness of the functions included in the digital safety plan.

The findings of this study have important clinical and practical implications. The observed association between safety plan activation and reduced ED readmission for suicidal behavior underscores the potential of digital safety plans in secondary suicide prevention. Integrating such tools into routine postsuicide attempt care could provide an accessible, scalable, and potentially cost-effective intervention to mitigate future crises. Moreover, digital safety plans offer an ecologically valid approach by enabling real-time usage monitoring and empowering individuals with digital tools and suicide-related coping strategies to manage future suicidal crises [24].

Digital mental health interventions have the potential to leverage technology to increase user engagement in suicide prevention and ensure continuity of treatment between emergency or hospital services and outpatient care [37]. Furthermore, digital interventions could be cost-effective, given their promising cost-benefit ratio in suicide prevention within the framework of current mental health interventions, which could support increased public health policy investment in digital strategies [38,39]. In particular, digital safety plans for suicide prevention present a compelling alternative to traditional methods, offering enhanced usability and reduced access barriers. These plans can integrate multimedia elements such as personal photos, prerecorded messages, relaxation videos, and links to health resources [19]. Their immediate availability, customizable nature, and dynamic functionality—allowing for real-time updates—further distinguish them [26].

However, despite these advantages, the practical usability of digital safety plan outside clinical environments remains underexplored. Enhancing accessibility and monitoring use are crucial for understanding their preventive impact. Research indicates that many individuals seeking suicide-related support are unfamiliar with safety plans or lack these in their treatment plans [17]. Furthermore, depressive moods and a perceived lack of collaborative planning with clinicians may hinder effective use [18]. Future studies should prioritize the sustainability of these tools, focusing on identifying usage barriers and implementing user-driven improvements through rigorous follow-up. Integrating specialized telephone follow-up could also provide timely support and reinforce user engagement by offering guidance on coping strategies [14].

### Limitations

First, the generalizability of our findings is limited by the small sample size, which was largely composed of women and did not include adolescents. Second, this study lacked detailed data on the duration of the use for each digital safety plan function and its perceived utility. Such data could provide valuable insights into which specific functions, or their combinations, are associated with the reduction of suicidal ideation and ED visits. Third, we did not collect information on participants who did not use or activate the digital safety plan. Understanding why some individuals did not engage with the tool (eg, due to perceived lack of need, usefulness, or technological barriers) would be valuable. Fourth, the absence of a control group limited our ability to directly compare the efficacy of digital versus traditional safety plan in suicide prevention. Finally, data on suicidal behavior features were based on baseline and self-reported measures. Future research could enhance these findings by incorporating real-time, in-app monitoring of suicidal ideation (eg, intensity and duration) and correlating it with digital safety plan use.

### Conclusions

To our knowledge, this is the first study to demonstrate that activating a digital safety plan can reduce the likelihood of repeat suicide-related ED visits by 50%. While these results do not directly demonstrate a decrease in suicidal behavior among participants, the reduction in ED visits following an index suicidal event suggests that individuals may find supportive elements to manage a crisis by readily accessing suicide-related coping strategies through the activation of digital safety plan. This suggests that digital safety plans may serve as a valuable resource, as a complement to interventions delivered in the ED. Future research should investigate the dynamics of digital safety plan use among participants, focusing on which functions (ie, tabs) are most frequently used and their specific associations with reductions in suicidal behavior and ED visits.

## Abbreviations

C-SSRS: Columbia Suicide Severity Rating Scale
ED: emergency department
EMI: ecological momentary intervention
SPI: safety planning intervention
